# A double-blind placebo-controlled trial of minocycline on translocator protein distribution volume in treatment-resistant major depressive disorder

**DOI:** 10.1038/s41398-021-01450-3

**Published:** 2021-05-29

**Authors:** Sophia Attwells, Elaine Setiawan, Pablo M. Rusjan, Cynthia Xu, Stephen J. Kish, Neil Vasdev, Sylvain Houle, Apitharani Santhirakumar, Jeffrey H. Meyer

**Affiliations:** 1 Brain Health Imaging Centre and Campbell Family Mental Health Research Institute at the Centre for Addiction and Mental Health, 250 College Street, Toronto, ON M5T 1R8 Canada; 2 Department of Pharmacology and Toxicology, University of Toronto, 1 King’s College Circle, Toronto, ON M5S 1A8 Canada; 3 Douglas Research Centre, McGill University, 6875 Boulevard Lasalle, Montreal, QC H4H 1R3 Canada; 4 Department of Psychiatry, University of Toronto, 250 College Street, Toronto, ON M5T 1R8 Canada

**Keywords:** Predictive markers, Predictive markers

## Abstract

Gliosis is implicated in the pathophysiology of many neuropsychiatric diseases, including treatment-resistant major depressive disorder (TRD). Translocator protein total distribution volume (TSPO V_T_), a brain marker mainly reflective of gliosis in disease, can be measured using positron emission tomography (PET). Minocycline reduces gliosis and translocator protein binding in rodents, but this is not established in humans. Here, the ability of oral minocycline to reduce TSPO V_T_ was assessed in TRD. To determine whether oral minocycline, as compared to placebo, can reduce prefrontal cortex (PFC), anterior cingulate cortex (ACC), and insula TSPO V_T_ in TRD, twenty-one TRD participants underwent two [^18^F]FEPPA PET scans to measure TSPO V_T_. These were completed before and after either oral minocycline 100 mg bid or placebo which was administered in a randomized double-blinded fashion for 8 weeks. There was no significant difference between the minocycline and placebo groups on change in TSPO V_T_ within the PFC, ACC, and insula (repeated measures ANOVA, effect of group interaction, PFC: *F*_1,19_ = 0.28, *P* = 0.60; ACC: *F*_1,19_ = 0.54, *P* = 0.47; insula *F*_1,19_ = 1.6, *P* = 0.22). Oral minocycline had no significant effect on TSPO V_T_ which suggests that this dosage is insufficient to reduce gliosis in TRD. To target gliosis in TRD either alternative therapeutics or intravenous formulations of minocycline should be investigated. These results also suggest that across neuropsychiatric diseases in humans, it should be assumed that oral minocycline will not reduce TSPO V_T_ or gliosis unless empirically demonstrated.

## Introduction

In the pathophysiology of several burdensome, frequently treatment-resistant neuropsychiatric diseases, including Alzheimer’s disease, Parkinson’s disease, Huntington’s disease, obsessive compulsive disorder, and major depressive disorder (MDD), an inflammatory response of a gliosis composed of activation and proliferation of microglia and/or astroglia is implicated^1,2^. Presently there is no established intervention for targeting this gliosis in humans, but minocycline reduces brain gliosis in rodent and in vitro studies. Minocycline is a second-generation tetracycline antibiotic with off target anti-inflammatory effects. The anti-inflammatory effects include lowering of lipopolysaccharide (LPS) induced brain expression of major histocompatibility complex II, tumor necrosis factor ɑ, interferon γ, interleukin 6, and interleukin IL-1β mRNA and/or protein; and reduction of microglial and astroglial activation and proliferation in animal disease models of neurodegeneration, traumatic brain injury, and sickness behavior^3–7^. Although these models suggest that minocycline has an important translational application to target gliosis, it has not been definitively demonstrated that minocycline affects gliosis in human neuropsychiatric diseases.

Translocator protein (TSPO) is expressed in activated microglia, activated astroglia, and endothelial cells. In rodent models of gliosis after LPS administration, toxin, and stroke, overall TSPO binding is strongly related to its magnitude of expression in activated microglia^8–10^. In human neuropsychiatric illnesses, such as AD, HIV encephalitis, multiple sclerosis, amyotrophic lateral sclerosis, frontotemporal dementia, and stroke, TSPO is expressed mainly in activated microglia and to a lesser extent in astroglia^11–13^. In health, most TSPO expression is consequent to its presence in endothelial cells. Thus the differential elevation of TSPO binding in neuropsychiatric disease as compared to health is usually attributed to the greater activation and proliferation of TSPO-positive microglia and astroglia that occur during gliosis^2,14^.

Minocycline reduces TSPO binding in rodents with gliosis but in humans there has been minimal study. In rats, the heightened TSPO binding associated with conditions that increase microglial and astroglial activation like middle cerebral artery occlusion, toll-like receptor 2 agonist exposure, and pilocarpine/kainate-induced status epilepticus are reduced by minocycline administration with substantial effects of 30–50%^15–17^. The first clinical trial of minocycline with TSPO PET imaging in humans had a sample size too small to be analyzed statistically with only three cases of multisystem atrophy receiving minocycline^18^. The other trial evaluating the effect of oral minocycline on TSPO binding in humans was conducted in traumatic brain injury and it compared the effect of minocycline in seven cases to placebo in five cases, finding a significant 20% reduction of TSPO distribution volume (TSPO V_T_), an index of TSPO density, in gray matter and white matter regions as measured with [^11^C]PBR28 PET^19^. The magnitude of effect in the latter study is promising. However, it would have been ideal to have included a few more cases in each group and it is unclear whether this result is generalizable to other psychiatric diseases.

In the present study, we evaluated the effect of minocycline on TSPO V_T_ as measured with [^18^F] FEPPA PET in treatment-resistant major depressive episodes (MDE) of MDD^14^. [^18^F]FEPPA has excellent properties including high, selective affinity for TSPO^20^, increased binding during induced neuroinflammation^21^, no detectable radioactive metabolites in brain in preclinical assessment^22^ and a high ratio of specific binding relative to free and non-specific binding^22,23^. TSPO PET imaging studies of MDE from MDD consistently report greater TSPO V_T_, or similar measures of TSPO binding, in the prefrontal cortex (PFC) and anterior cingulate cortex (ACC), as demonstrated across six different studies at four different sites totaling 142 patients and 93 controls^24–28^, with ACC values elevated 15–67% and PFC values elevated 25–35%. One additional [^11^C]PBR28 study reported a negative result within a sample combining five MDE subjects and four recovered depressed subjects, however, the same group reports that the five subjects in a current MDE also had elevated TSPO V_T_ values in gray-matter regions^29^.

Two double-blind, randomized placebo-controlled trials of minocycline demonstrated promising effects on MDE with one trial demonstrating a significant reduction on core symptoms and the other reporting trend reductions in anxiety^30,31^. Therefore, given that minocycline reduces gliosis^3–7^ and TSPO binding in rodent models^15–17^, that there is some evidence of symptom reduction in double-blind placebo-controlled clinical trials of minocycline in MDE^30,31^, and that TSPO V_T_ is consistently elevated in the PFC, ACC and insula during MDE^24–28^, it was hypothesized that minocycline would lower TSPO V_T_ in the PFC, ACC, and insula in MDE in humans. The PFC and ACC were prioritized regions of interest (ROIs) because neuropathological abnormalities are frequently identified within these regions in MDE, subregions within these structures regulate affect, and these regions are functionally sensitive to depressed mood induction^32^. In addition, the insula was prioritized because it is implicated in mediating some of the sickness behaviors in MDD: The insula participates in homeostatic regulation and interoceptive signaling;^33,34^ and has elevated TSPO V_T_ during MDE^27,28^.

## Materials, participants, and methods

### Participants

**Fig. 1:**
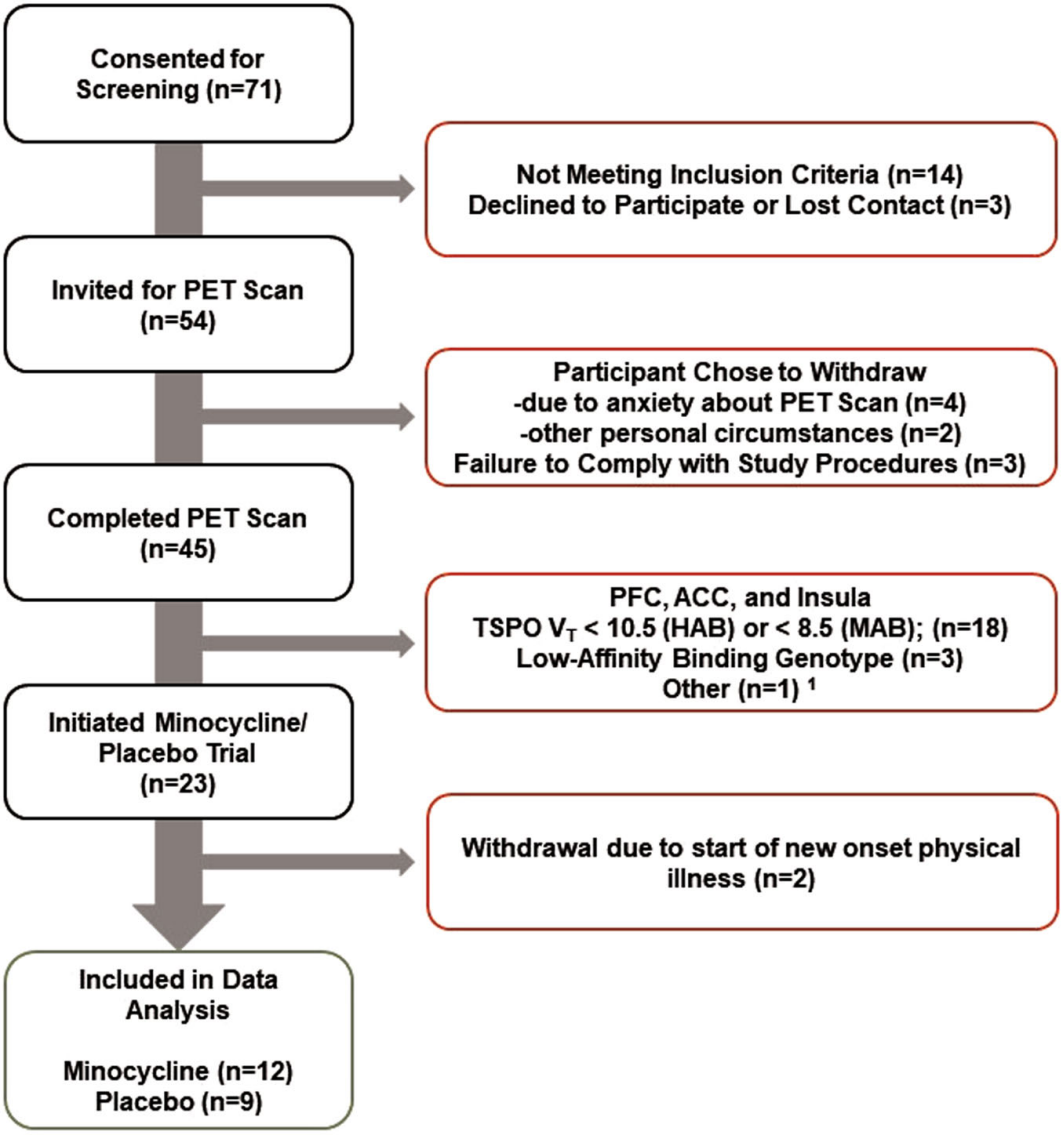
Flow diagram of study participants. Flow of participants through the study.^1^ Diagnosis reclassified as bipolar type II. ACC anterior cingulate cortex, PFC prefrontal cortex, PET positron emission tomography, TSPO V_T_ translocator protein total distribution volume.

Twenty-three participants with treatment-resistant MDE secondary to MDD were recruited from the Greater Toronto Area and a tertiary psychiatric hospital, Centre for Addiction and Mental Health (CAMH), between February 2015 and March 2019 (Fig. 1, Table 1). Inclusion criteria included age 18–65; a positive diagnosis of MDE based on the Structured Clinical Interview for DSM-IV with confirmation by a consultation with a psychiatrist (J.H.M.) and a minimum score of 19 on the 17 item Hamilton Depression Rating Scale (HDRS) at screening^35^. TRD was defined as a history of non-response to a clinically appropriate dose and duration of at least one antidepressant whose mechanism of action includes raising extracellular serotonin concentration and one or more antidepressants whose mechanism of action includes raising norepinephrine concentration; or non-response to at least one serotonin and norepinephrine reuptake inhibitor. All participants confirmed receiving a stable clinical dose of an antidepressant medication for at least 4 weeks prior to PET scanning (see Supplementary Table 1).

**Table 1:** Demographic characteristics of study participants.

Characteristic	Minocycline (*n* = 12)	Placebo (*n* = 9)	*P* value
Sex			0.66^a^
Female	8	7	
Male	4	2	
Translocator protein genotype^b^			1.000^a^
High-affinity binders	9	6	
Mixed-affinity binders	3	3	
Age, years, mean (SD)	36.5 (13.4)	36.9 (12.0)	0.94^c^
Body mass index, mean (SD)	24.7 (2.7)	25.1 (4.3)	0.76^c^
Level of education, years, mean (SD)	17.3 (4.0)	15.3 (2.3)	0.22^c^
HDRS score^d^	20.2 (3.5)	20.3 (6.1)	0.94^c^
Age at first MDE, years, mean (SD)	14.8 (6.3)	20.4 (11.0)	0.15^c^
Number of MDEs, mean (SD)	5.4 (3.4)	8.0 (5.6)	0.21^c^
Number of previous antidepressant treatment, mean (SD)^e^	5.0 (3.4)	5.6 (2.0)	0.67^c^
Previous antidepressant treatment, years, mean (SD)	9.2 (7.1)	7.3 (6.5)	0.53^c^
Years of untreated major depressive disorder, mean (SD)	11.9 (10.5)	8.9 (8.2)	0.49^c^
Number of previous suicide attempts, mean (SD)	0.3 (0.6)	0.4 (0.7)	0.52^c^

*HDRS* Hamilton Depression Rating Scale, *MDE* major depressive episode, *PET* positron emission tomography, *SD* standard deviation, [^18^F]FEPPA, F 18-labeled N-(2-(2-fluoroethoxy)benzyl)-N-(4-phenoxypyridin-3-yl)acetamide).

^a^Fisher’s exact test.

^b^rs6971 genotype influences the binding of second-generation radiotracer ligands including [^18^F]FEPPA.

^c^Univariate analysis of variance.

^d^Score derived at baseline PET scan. At the time of initial screening, HDRS score was 19 or greater but with ongoing variation in severity of illness, five participants had HDRS below 19 at the time of the first PET scan.

^e^Previous treatments included quetiapine, repetitive transcranial magnetic stimulation, magnetic seizure therapy, and electroconvulsive therapy.

Participants were excluded if they had a history of, or had concurrent alcohol or substance dependence. This was corroborated by urine drug testing at screening and on the PET scan day. In addition, to avoid potential biases from recent infection, all were free from acute medical illnesses for the 2 weeks before PET scanning. Also, participants were excluded if they had used brain stimulation treatments within the 6 months before scanning, had used anti-inflammatory drugs lasting at least 1 week within the past month, or were taking hormone replacement therapy. Other exclusion criteria were bipolar disorder (type I or II) and antisocial personality disorder. No participant had a history of neurological illness, autoimmune disorder, severe hepatic or renal disease, gastrointestinal disease, ischaemic heart disease, cerebrovascular disorder, or congestive heart failure. Participants were also excluded if they were pregnant or breastfeeding; and all women received a urine pregnancy test on the PET scan day.

Like all second-generation TSPO radioligands, the binding affinity of [^18^F]FEPPA for TSPO is affected by a single-nucleotide polymorphism (rs6971; C→T) in exon 4 of the *TSPO* gene (NCBI Entrez Gene 706)^36,37^. High-affinity binders (HAB) express a single high-affinity binding site for TSPO, whereas mixed-affinity binders (MAB) express in equal quantities both high-affinity and low-affinity binding sites for TSPO. Previous studies demonstrate the rs6971 polymorphism to be a significant predictor of [^18^F]FEPPA TSPO V_T_ in human brains^36^. Individuals with high-affinity binding (Ala147/Ala147) and mixed-affinity binding (Ala147/Thr147) account for more than 95% of the population in the Greater Toronto Area^28,36^. Intravenous whole blood was collected and the rs6971 polymorphism was genotyped (see Supplementary Information). Since the translational aim for the clinical setting was to develop minocycline for those with more prominent gliosis, an additional inclusion criterion was that TSPO V_T_ was at least above 10.5 for HAB and 8.5 for MAB in any of the primary ROIs (PFC, ACC, insula). By applying such a threshold, it was anticipated that the resulting TSPO V_T_ values in the sample gathered would ensure recruitment of MDE participants with TSPO V_T_ values higher than most healthy people^28^. Homozygotes for the low-affinity binding gene (Thr147/Thr147) were withdrawn from the study.

This study was approved by the Research Ethics Board of the CAMH and was therefore performed in accordance with the ethical standards established in the 1964 Declaration of Helsinki. All participants gave their informed consent prior to their inclusion in the study. All details that might disclose the identity of the participants have been omitted.

### Image acquisition and analysis

Each participant underwent two [^18^F]FEPPA PET scans (HRRT; CPS/Siemens, Knoxville, TN, USA): one scan pre minocycline-placebo treatment and one scan post 8-week minocycline-placebo treatment. Intravenous [^18^F]FEPPA was administered as a bolus (PET Scan 1: mean [standard deviation (SD)], 188.2 [12.5] MBq; PET scan 2: mean [SD], 186.0 [12.5] MBq). The [^18^F]FEPPA was of high radiochemical purity (PET scan 1: >99.0%; PET scan 2: 99.3%) and high specific activity (PET scan 1: mean [SD], 65.8 [41.3] TBq/mmol; PET scan 2: mean [SD], 145.0 [146.2] TBq/mmol (no relationship between TSPO V_T_ and specific activity; *R*^2^ < 0.012; see Supplementary Results). The PET scan duration was 125 min after the injection of [^18^F]FEPPA (see Supplementary Information).

Primary ROIs were the PFC, ACC, and insula. Additional regions sampled included subregions of the PFC (medial, ventrolateral, dorsolateral, and orbitofrontal), temporal cortex, parietal cortex, occipital cortex, thalamus, dorsal putamen, dorsal caudate, ventral striatum, and hippocampus. ROIs were defined as previously described^38^. ROIs were delineated on proton density magnetic resonance imaging scans (Signa 3-T MRI scanner, General Electric, Milwaukee, WI, USA; section thickness 2 mm, repetition time 6000 ms, echo time 8 ms, flip angle 90°, one excitation, acquisition matrix 256 × 192, and field of view 16.5 cm) using the in-house software, Regions of Mental Interest. Arterial sampling was conducted to determine the input to a two tissue compartment model to measure TSPO V_T_ (see Supplementary Information).

### Minocycline dosing regime and compliance calculation

All participants received either minocycline or placebo for 8 weeks in a randomized, double-blind, placebo-controlled trial. Randomization was performed using an algorithm created in R Version 3.1.0 in February 2015. The algorithm consisted of a 1:1 ratio of active vs. placebo. Bloc randomization was used (bloc size = 20), the total number of codes was 80, and drop outs were not replaced. For placebo, the number and appearance of the pills were identical to minocycline. Dosing of minocycline was 50 mg per day on week 1, 50 mg bid on week 2, and 100 mg bid on weeks 3–8. Minocycline taken 100 mg bid is well tolerated in human clinical trials^30,31^ and was the same dose given by Scott et al. which lowered TSPO V_T_ in traumatic brain injury^19^. For tapering, dosing was reduced to 50 mg bid for a week, and then stopped. The blind was broken following the completion of 20 participants and 1 additional participant completed the study in a blinded state. Participants attended follow-up appointments with the treating psychiatrist and research staff at weeks 2, 4–5, and 8.

To increase compliance, minocycline and placebo were given to the participants in blister packs. The blister packs contained designated sealed compartments for the medication to be taken at times of the day (Monday through Sunday, breakfast and supper). To calculate compliance, participants informed study staff if they missed a dosage. In addition, blister packs were collected after use, and remaining pills were counted and returned to the CAMH pharmacy. Compliance was recorded in a subset of 14 subjects and average compliance was calculated to be 99.3% (SD = 1.6).

### Mood questionnaires

The 17 item HDRS was completed by participants during all study visits^35^. Baseline scores were taken from the day at which minocycline or placebo was initiated. Participants returned to the CAMH for follow-up visits at weeks 2 and 4–5. The second [^18^F]FEPPA PET scan took place during week 8 of minocycline or placebo treatment.

### Statistical analysis

For the primary analyses, a repeated measures analysis of variance (rmANOVA) was applied. One repeated measure was TSPO V_T_ within each region before and after the intervention (minocycline or placebo), a measure to assess change over the time of the intervention. The other repeated measure was region (PFC, ACC, or insula) which was sampled concurrently at each timepoint within each participant. Hence the within group factors were region and scan time (first or second PET scan) and the between group factors were minocycline or placebo with the interaction between scan time and group being the key analysis. Similar secondary analyses were conducted with a rmANOVA for each region, with regional TSPO V_T_ as the dependent variable, scan time and group as the predictor variables, assessing the interaction between scan time and group. As an additional analysis, a mixed effects model was applied in which participants are considered random effects; brain regions and scan time are within-subject fixed effects; group (minocycline or placebo) is a between subject fixed effect; and the interaction between visit and group was assessed.

Additional exploratory analyses were conducted to assess the relationship between change in the severity of MDE and change in TSPO V_T_ within the PFC, ACC, and insula. The two TSPO V_T_ values for each region from the first and second [^18^F]FEPPA PET scans were the repeated dependent variables and then the change in HDRS and the rs6971 genotype were applied as the independent variables, prioritizing the interactions between change in HDRS and scan time.

## Results

### Participant demographics and measures at baseline

Overall, 23 TRD eligible participants were recruited and 21 completed the 8-week trial (Fig. 1). Twelve participants received minocycline and nine received placebo (Table 1). The mean and SD of the age of the participants, age of first MDE onset, and number of previous non-responses to treatment were 36.7 ± 12.5, 17.2 ± 8.8, and 5.2 ± 2.8, respectively. Assigned sex at birth of participants included 15 females and 6 males. Preferred genders of participants included 15 females and 6 males. Age, sex, rs6971 genotype, BMI, and HDRS score at the time of the first PET scan were similar across groups as were other demographic characteristics (Table 1). Baseline TSPO V_T_ values in all regions assessed were similar between the minocycline and placebo groups (analysis of variance (ANOVA), *F*_1,18_ = 3.4–0.27, *P* = 0.081–0.61; baseline TSPO V_T_ values displayed in Table 2).

**Table 2:** Effect of oral minocycline treatment on regional translocator protein total distribution volume.

	Baseline TSPO V_T_		Post-treatment TSPO V_T_		^a^Change in TSPO V_T_			
	Minocycline (*n* = 12)	Placebo (*n* = 9)	Minocycline (*n* = 12)	Placebo (*n* = 9)	Minocycline (*n* = 12)	Placebo (*n* = 9)	Effect of treatment group^b^	
	Mean (SD)	Mean (SD)	Mean (SD)	Mean (SD)	Mean (SD)	Mean (SD)	*F*_1,19_	*P* value
PFC	12.4 (2.6)	13.6 (4.9)	11.8 (3.6)	12.5 (3.7)	−0.61 (1.7)	−1.1 (2.5)	0.28	0.60
MPFC	12.2 (2.6)	13.3 (5.1)	11.3 (3.8)	11.9 (3.7)	−0.92 (1.9)	−1.4 (2.3)	0.24	0.63
VLPFC	13.2 (2.5)	14.6 (5.0)	12.8 (4.0)	13.5 (4.2)	−0.38 (2.0)	−1.2 (2.7)	0.58	0.46
DLPFC	12.4 (2.7)	13.4 (4.7)	11.8 (3.5)	12.4 (3.7)	−0.58 (1.8)	−0.97 (2.3)	0.19	0.67
OFC	12.3 (3.4)	14.1 (4.3)	11.4 (3.9)	12.0 (3.4)	−0.83 (2.0)	−2.1 (2.4)	1.8	0.19
ACC	12.1 (3.0)	13.2 (5.0)	11.5 (4.4)	11.9 (3.3)	−0.51 (2.8)	−1.3 (2.6)	0.54	0.47
Insula	12.9 (3.0)	14.1 (4.7)	12.0 (3.9)	12.1 (3.8)	−0.93 (1.7)	−2.0 (2.2)	1.6	0.22
Temporal cortex	12.6 (3.0)	13.8 (5.0)	12.1 (4.1)	12.1 (3.4)	−0.53 (1.6)	−1.8 (2.2)	2.3	0.15
Parietal cortex	13.2 (3.0)	14.6 (5.4)	13.0 (3.9)	13.2 (4.2)	−0.21 (1.9)	−1.4 (2.3)	1.7	0.21
Occipital cortex	12.7 (3.0)	13.8 (5.1)	12.0 (4.5)	12.2 (3.8)	−0.69 (1.8)	−1.6 (2.6)	0.94	0.35
Thalamus	15.1 (3.8)	16.0 (5.3)	14.2 (5.3)	13.8 (4.4)	−0.89 (2.5)	−2.2 (3.4)	0.99	0.33
Dorsal putamen	10.9 (2.8)	11.7 (4.1)	10.2 (3.4)	10.2 (3.1)	−0.77 (1.7)	−1.5 (2.1)	0.72	0.41
Dorsal caudate	10.0 (2.4)	11.1 (3.9)	9.8 (3.2)	9.8 (3.4)	−0.25 (2.2)	−1.3 (2.7)	0.95	0.34
Ventral striatum	11.8 (3.3)	12.1 (4.8)	10.7 (3.9)	10.9 (3.2)	−1.1 (2.3)	−1.2 (2.6)	0.02	0.89
Hippocampus	11.2 (3.0)	13.1 (5.3)	11.4 (4.6)	11.6 (3.3)	0.20 (3.4)	−1.5 (5.1)	0.81	0.38

*ACC* anterior cingulate cortex, *DLPFC* dorsolateral prefrontal cortex, *MPFC* medial prefrontal cortex, *OFC* orbitofrontal cortex, *PFC* prefrontal cortex, *PET* positron emission tomography, *SD* standard deviation, *TSPO V*_*T*_ translocator protein total distribution volume, *VLPFC* ventrolateral prefrontal cortex.

^a^Change in TSPO V_T_ was calculated as regional TSPO V_T_ post-treatment minus regional TSPO V_T_ baseline. Analysis of variance comparing baseline TSPO V_T_ values found no significant difference between groups for any regions (effects of group and genotype included, effect of group, *F*_1,18_ = 3.4–0.27, *P* = 0.081–0.61).

^b^Repeated measures analysis of variance evaluating effect of group interaction on repeated measure of TSPO V_T_.

### No effect of minocycline vs. placebo on change in TSPO V_T_

**Fig. 2:**
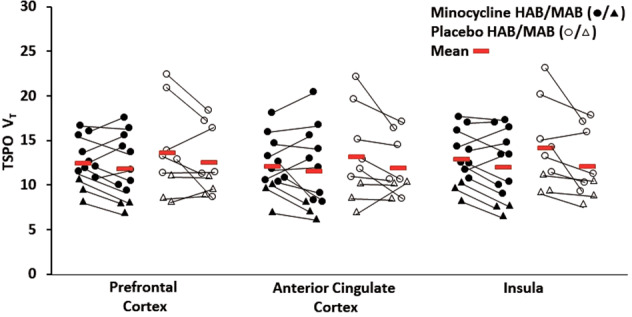
Effect of oral minocycline treatment on translocator protein total distribution volume in participants with treatment-resistant major depressive disorder. There were no significant differences in prefrontal cortex, anterior cingulate cortex, or insula TSPO V_T_ between pre- and post-minocycline treatment in the group receiving minocycline as compared to the group receiving placebo (repeated measures ANOVA, effect of group interaction, PFC: *F*_1,19_ = 0.28, *P* = 0.60; ACC: *F*_1,19_ = 0.54, *P* = 0.47; insula *F*_1,19_ = 1.6, *P* = 0.22). All second-generation TSPO radioligands, such as [^18^F]FEPPA, show differential binding according to the single-nucleotide polymorphism rs6971 of the TSPO gene, resulting in high-affinity binders (circles) and mixed-affinity binders (triangles). TSPO V_T_ values represent raw values unadjusted for genotype. ANOVA analysis of variance, HAB high-affinity binder, MAB mixed-affinity binder, TSPO V_T_ translocator protein total distribution volume, [^18^F]FEPPA, F 18-labeled *N*-(2-(2-fluoroethoxy)benzyl)-*N*-(4-phenoxypyridin-3-yl)acetamide).

There was no differential effect between the minocycline and placebo groups on change in TSPO V_T_ across the first and second PET scans (scan time) in the PFC, ACC, and insula (rmANOVA; first or second TSPO V_T_ and region were repeated dependent variables with first or second TSPO V_T_ reflecting the effect of scan time; interaction effect of group on scan time; *F*_1,19_ = 0.79, *P* = 0.39). Nor was there a differential effect between minocycline and placebo groups on change in TSPO V_T_ over time within individual regions. (rmANOVA; first or second TSPO V_T_ repeated dependent variables analyzed within each region, PFC: *F*_1,19_ = 0.28, *P* = 0.60; ACC: *F*_1,19_ = 0.54, *P* = 0.47; insula *F*_1,19_ = 1.6, *P* = 0.22); Fig. 2). When genotype was added as an additional independent variable it was not significant as a predictor of the repeated measure and then not included in the analyses. There was also no differential effect of minocycline vs. placebo on change in TSPO V_T_ in any other region (rmANOVA, applied for each region, first or second TSPO V_T_ was the repeated dependent variable reflecting scan time, interaction of group on change in TSPO V_T_; *F*_1,19_ = 2.3–0.02, *P* = 0.15–0.89; Table 2). When genotype was added as an additional independent variable it was not significant as a predictor of the repeated measure and then not included in the analyses. In addition, when a mixed effects model was applied, with participant as the random effect, repeated scan as a fixed effect, region as a fixed effect, and group as a fixed effect, there was no significant interactive effect between the effects of repeated scan and group (*P* = 0.39).

### No significant relationship between change in TSPO V_T_ and change in hamilton depression rating scale scores

Mean change in HDRS was modest in both groups (mean [SD]: minocycline −5.7 [7.6]; placebo −7.1 [5.6]; with three participants in the placebo group and two participants in the minocycline group responding (HDRS decrease ≥50%), and two participants in the placebo group and one participant in the minocycline group remitting (final HDRS ≤ 7). There was no significant difference in the reduction in HDRS between groups (mixed effects model, *P* = 0.64, see Supplementary Information)

**Fig. 3:**
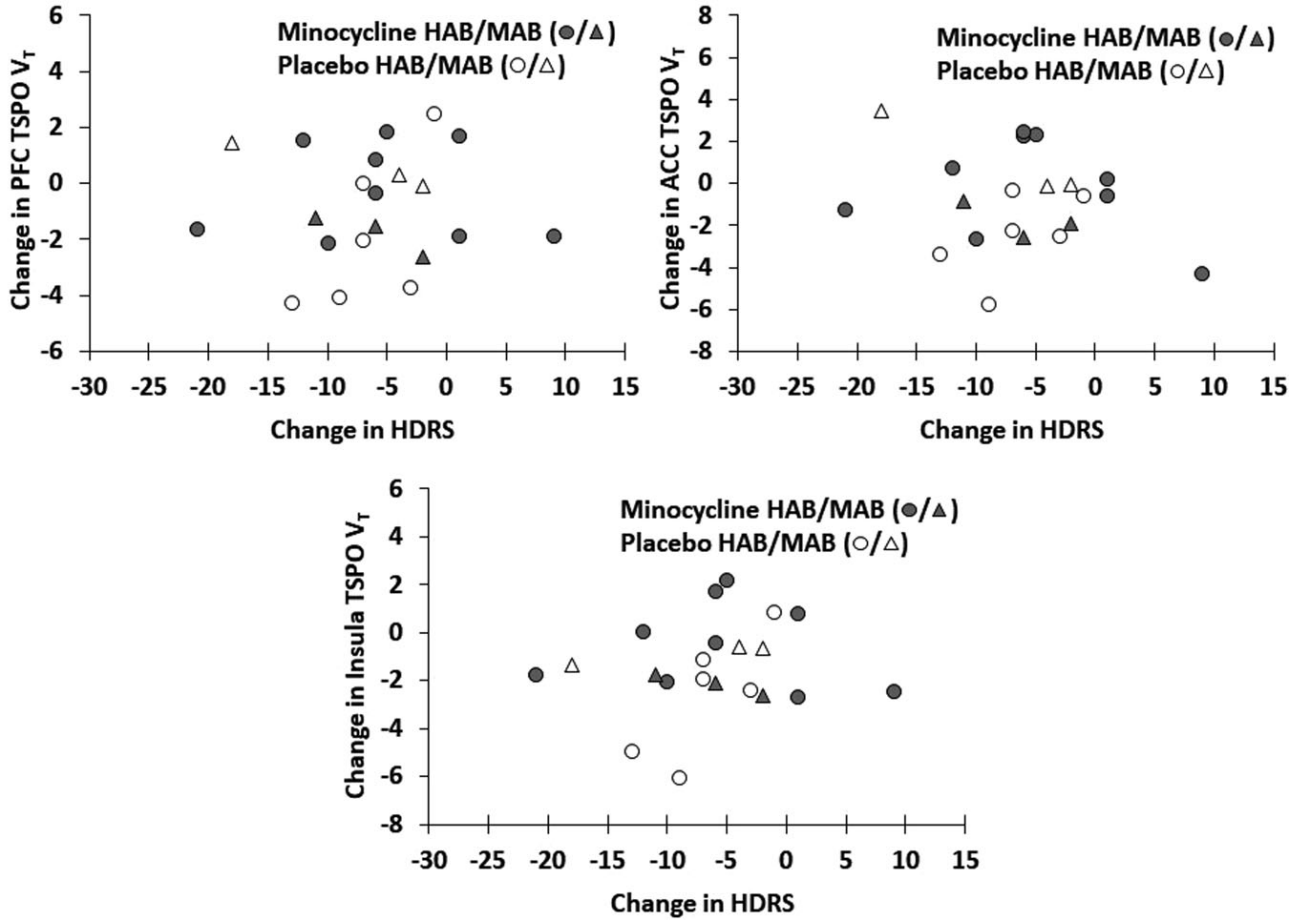
Relationship between change in regional translocator protein total distribution volume and change in hamilton depression rating scale scores. There were no significant relationships between the change in prefrontal cortex, anterior cingulate cortex, or insula TSPO V_T_ with change in HDRS scores (repeated measures ANOVA, TSPO V_T_ as dependent variable, effect of HDRS change as covariate, *F*_1,19_ = 0.66–0.06, *P* = 0.43–0.82). Similarly, nonsignificant effects were observed within the minocycline treated group alone. ACC anterior cingulate cortex, HAB high-affinity binder, HDRS Hamilton Depression Rating Scale, MAB mixed-affinity binder, PFC prefrontal cortex, TSPO V_T_ translocator protein total distribution volume.

There was no relationship between change in the severity of MDE and change in TSPO V_T_ within the PFC, ACC, and insula (rmANOVA for each region; scan time of first or second TSPO V_T_ were repeated dependent variables; effect of HDRS *F*_1,19_ = 0.66–0.06, *P* = 0.43–0.82; Fig. 3). Additional analyses assessing the interaction of HDRS and genotype or inclusion of genotype were similarly nonsignificant. Repeating the same analysis within the minocycline group alone similarly found no relationship between severity of MDE and change in TSPO V_T_ (rmANOVA for each region; scan time of first or second TSPO V_T_ were repeated dependent variables; effect of HDRS *F*_1,10_ = 0.54–0.02, *P* = 0.48–0.89). Additional analyses assessing the interaction of HDRS and genotype or inclusion of genotype were similarly nonsignificant.

## Discussion

This study demonstrated no significant effect of minocycline as compared to placebo on TSPO V_T_ in the PFC, ACC, insula, or any other brain region assayed in treatment-resistant MDE. This has important implications for repurposing minocycline both in MDD and in human neuropsychiatric diseases. While the results appear to differ from previous rodent studies and the one previous study in humans, this may be accounted for by differences in the experimental conditions and/or participants, demonstrating the importance of empirical testing of target engagement.

The lack of effect of minocycline on TSPO V_T_ may be interpreted as a lack of effect on TSPO density, which, in neuropsychiatric diseases mainly reflects gliosis with some additional contribution from endothelial cells^2,14^. This argues that minocycline had no effect on these sources of TSPO density in the brain at a dose of 100 mg bid, which is considered a maximum well-tolerated oral dose^30,31^. This argues that repurposing minocycline for MDE in a clinical setting by administering a course of treatment with the aim of obtaining a sustained reduction in gliosis is unlikely. It also suggests the rationale for its use in clinical trials should be oriented towards alternative mechanisms like protecting against apoptosis through inhibiting caspase 1 and 3, and upregulating Bcl2 expression; engagement of potential downstream effects of gliosis like inhibiting inducible nitric oxide synthase or COX-2; or other neuroprotective effects, such as scavenging peroxynitrites or inhibiting matrix metalloproteinases^4^.

The differences in the effect of minocycline in the present study with others may be accounted for by differences in the experimental conditions. Minocycline suppresses elevations in TSPO binding associated with gliosis and reduces gliosis itself in rodents^15–17,39^. However, the dosing of minocycline in such studies, is 3–30 times larger relative to body mass^39^. The results of the present study also contrast the previous human data of a 20% reduction in TSPO V_T_ reported across brain regions assessed in seven cases treated with minocycline vs. five receiving placebo, in TBI cases within 6 months of the injury^19^. While the present study is almost twice as large, another factor to consider is the duration of disease with the possible entrenchment of pro-inflammatory processes contributing and maintaining the TSPO V_T_ elevation. The mean duration of MDD illness in the present study was 20 years in contrast to a 6-month duration since TBI in the past study^19^ so the relative duration of illness differs by a factor as much as 40. Given the negative results of the present study and given that conditions underlying gliosis in each neuropsychiatric illness differ, rather than assuming target engagement of minocycline for gliosis or TSPO V_T_, it should be tested within specific illnesses.

The present study sampled 21 participants and it could be speculated as to whether increasing the sample size would alter the conclusions. This is unlikely given the differential change between groups and the SD of such changes for the PFC, ACC, and insula. The resulting confidence interval indicates there is a 95% chance that the magnitude of differential reduction in TSPO V_T_ favoring minocycline in these regions is less than 9%, 11%, and 3%, respectively. This is much smaller than the mean disease effect of MDE in these regions which is typically between 20 and 35% and similar to other neuropsychiatric diseases^2,40,41^.

This study has several limitations. First, while the elevations of TSPO in neuropsychiatric illnesses are associated with greater expression in microglia with some astroglial contributions, there is some expression of TSPO in endothelial cells. However, it seems unlikely that the endothelial cell component would obscure an effect of minocycline given that in neuropsychiatric diseases and inflammatory states, TSPO expression is most prominent in glial cells^8–13^; the spatial extent of endothelial cells is limited since they surround blood vessels and vascular contribution to PET is considered small representing ~5% of the input signal; the two tissue plus one compartment model proposed for modeling endothelial contributions is not advantageous for [^18^F]FEPPA PET^42^ and it seems unlikely that minocycline would evoke a strong opposite effect on TSPO expression in endothelial cells. Second, the dose of minocycline was limited to 100 mg which is the maximum well-tolerated oral dose. Future studies could consider intravenous administration which may increase plasma levels at least twofold or alternative therapeutics, such as P2X7 inhibitors, PGE4 inhibitors, or peroxisome proliferator gamma agonists^43–45^. Third, TSPO participates in multiple functions including cholesterol transport, mitochondrial respiration, and apoptosis and it is possible that in future study it will be determined that there are other mechanisms which influence TSPO expression. Fourth, oral administration of minocycline might also affect the balance of the gut microbiome which has influences on bodily inflammation and its influences on brain inflammation^46^, which may have obscured a direct effect of minocycline. Regardless, the overall effect of oral minocycline, even if such indirect influences occurred, had a negligible effect on TSPO V_T_ in brain.

In summary, to our knowledge, this is the first investigation evaluating the effect of minocycline on a measure of gliosis in the brain of humans with MDE. This is also the second study to assess the effect of minocycline on a measure of gliosis in brain in humans, applying a sample almost twice as large as the previous study which also measured TSPO V_T_. There were no differences between minocycline and placebo on TSPO V_T_. Moreover, the data argue that a meaningful reduction in TSPO V_T_ with minocycline treatment, as compared to the disease effect on TSPO V_T_ in MDE, is highly unlikely. The negative result of the present study also demonstrates that it cannot be assumed that the effects of minocycline on TSPO V_T_ observed in animals in which the dosing is much higher, can be extrapolated to all human conditions. Rather, it is important to empirically test target engagement in specific neuropsychiatric illnesses as underlying disease etiologies and durations vary. Future study may consider more potent interventions, such as intravenous dosing of minocycline, or alternative therapeutics to reduce TSPO V_T_ in MDE.

## Supplementary information

Supplemental Information
