# Supplementary material for: A double-blind placebo-controlled trial of minocycline on translocator protein distribution volume in treatment-resistant major depressive disorder
Source: Transl Psychiatry. 2021 May 29;11:334. doi: 10.1038/s41398-021-01450-3 (PMC8164633; doi:10.1038/s41398-021-01450-3)
Supplement: Supplementary file 1 — Supplemental Information [file 41398_2021_1450_MOESM1_ESM.docx]

**Supplemental Information**

Supplement to: Attwells S, Setiawan E, Rusjan PM, et al. A Double-Blind Placebo Controlled Trial of Minocycline on Translocator Protein Distribution Volume in Treatment Resistant Major Depressive Disorder. *Translational Psychiatry.* 2020.

**Supplementary Methods**

**Image Acquisition and Analysis**

**DNA Extraction and Polymorphism Genotyping**

**Supplementary Results**

**Comment on the Distribution of Data**

**Assessment of Relationship of Specific Activity to TSPO V_T_**

**Comparison of Clinical Effect of Minocycline Versus Placebo**

**Supplementary References**

**Supplementary Table 1**

**Supplementary Methods**

**Image Acquisition and Analysis**

Positron emission tomography (PET) scans were obtained using the Siemens three-dimensional high-resolution research tomograph scanner (Siemens, Knoxville, TN, USA), which measures radioactivity in 207 1.2 mm thick slices. Emission data was acquired for 125 minutes which were then reconstructed into 34 timeframes: 1 frame of variable length, 5 x 30, 1 x 45, 2 x 60, 1 x 90, 1 x 120, 1 x 210, and 22 x 300 seconds. All PET images were corrected for attenuation using a single photon point source, cesium 137 (half-life, 30.2 years; energy, 662 keV). Further, PET images were reconstructed using a filtered back-projection algorithm and a Hann filter at Nyquist cut-off frequency.^1^ Arterial blood was collected automatically for the first 22.5 minutes, following radiotracer injection, using the automatic blood sampling system (ABSS, Model PBS-101; Veenstra Instruments, Joure, Netherlands). Manual blood samples (7 mL) were also drawn at -5, 2.5, 7, 12, 15, 20, 30, 45, 60, 90, and 120 post radiotracer injection. Automatic and manual blood samples were obtained to determine the input function of [^18^F]FEPPA in plasma for the kinetic analysis. The manual samples were used to determine the blood to plasma ratios and the relative fraction of parent radioligand within plasma. A bi-exponential function was used to fit the blood-to-plasma ratios and a Hill function was used to fit the percentage of un-metabolized [^18^F]FEPPA. Then the input data, a metabolite corrected arterial plasma curve, was generated by the product of the dispersion corrected blood curve with the other two curves (blood to plasma ratio and percentage of parent radiotracer). Then a 2-tissue compartment model was applied with the input data and the time activity curves from each region of interest (ROI) to quantitate translocator protein total distribution volume (TSPO V_T_) with [^18^F]FEPPA PET.^1^

ROI delineation and analysis were performed using the in-house image analysis software [Regions of Mental Interest (ROMI); Research Imaging Centre, Centre for Addiction and Mental Health]. In brief, ROMI is based on the individualization of a set of standard ROIs using magnetic resonance imaging (MRI) scan co-registered with the PET image, followed by a step of gray-matter voxel selection, which incorporates the probability of gray-matter, white-matter, and cerebrospinal fluid based on the segmentation of the individual MRI scan.^2^ For the anatomical delineation of ROIs, all participants underwent a two-dimensional axial proton density MRI scan (Signa 3-T MRI scanner, General Electric, Milwaukee, WI, USA; section thickness 2 mm, repetition time 6000 ms, echo time 8 ms, flip angle 90°, one excitation, acquisition matrix 256 × 192, and field of view 16.5 cm).

**DNA Extraction and Polymorphism Genotyping**

Intravenous whole blood was collected on PET scan day to obtain genomic DNA using high salt extraction methods.^3^ The rs6971 polymorphism was genotyped using a TaqMan assay on demand C_2512465_20 (Applied Biosystems, Foster City, CA, USA). Polymerase chain reaction (PCR) reactions were performed on a GeneAmp PCR System 9700 (Applied Biosystems), followed by end-point plate read and allele calling using an ABI 7900 HT (Applied Biosystems).

**Supplementary Results**

**Comment on the Distribution of Data**

The difference in translocator protein total distribution volume (TSPO V_T_) before and after minocycline or placebo was normally distributed in the prefrontal cortex (PFC), anterior cingulate cortex (ACC) and insula (INS) (Shapiro-Wilk, df =21, p=0.41, 0.92 and 0.50 respectively). There were no significant differences in the variances for TSPO V_T_ between the minocycline and placebo groups within the PFC, ACC or INS (F-test for comparison, p>0.05 for all comparisons).

**Assessment of Relationship of Specific Activity to TSPO V_T_**

The correlations between TSPO V_T_ and specific activity were evaluated within each main region of interest (PFC, ACC, and INS) within high affinity binders (HAB, n=30 [^18^F] FEPPA PET scans) and mixed affinity binders (MAB, n=12 [^18^F] FEPPA PET scans) sampling TSPO V_T_ values from all PET scans. There was no relationship between specific activity and TSPO V_T_ for each region (PFC: HAB r^2^=0.0016; MAB r^2^=0.012; ACC: HAB r^2^=0.0003; MAB r^2^=0.003; insula: HAB r^2^=0.009; MAB r^2^=0.01)

**Comparison of Clinical Effect of Minocycline Versus Placebo**

As an additional exploratory analysis, a repeated measures analysis using mixed effects model was applied with the HDRS values, evaluating the contribution of being in either the minocycline or placebo group on the repeated measure of the Hamilton Depression Rating Scale (HDRS). The effect was not significant (*P*=0.64). A repeated measures ANOVA with the same dependent variables and independent factor yielded similar results (effect of group, F_1,19_=0.23, *P*=0.64).

**Supplementary References**

1. Rusjan P.M. et al*.* Quantitation of translocator protein binding in human brain with the novel radioligand [18F]-FEPPA and positron emission tomography. *J. Cereb. Blood Flow Metab.* **31**, 1807-1816 (2011).
2. Rusjan P. et al*.* An automated method for the extraction of regional data from PET images. *Psychiatry Res.* **147**, 79-89 (2006).
3. Lahiri D. K. & Nurnberger J. I. Jr. A rapid non-enzymatic method for the preparation of HMW DNA from blood for RFLP studies. *Nucleic Acids Res.* **19**, 5444 (1991).

| **Supplementary Table 1: Antidepressants Taken Throughout Celecoxib Trial** | | |
| --- | --- | --- |
| **Participant Order^a^** | **Current Antidepressant Treatment** | **Duration of Treatment^b^** |
| 1 | Duloxetine | 1 month |
| 2 | Venlafaxine | 5 years |
| 3 | Clomipramine | 4-5 months |
| 4 | Citalopram  Bupropion | 2 years  2 years |
| 5 | Vortioxetine  Bupropion | 6 months  >2 months |
| 6 | Duloxetine | 2-3 months |
| 7 | Sertraline  Bupropion | 4 months  1 month |
| 8 | Escitalopram | 1.5 months |
| 9 | Desvenlafaxine | 8.5 months |
| 10 | Sertraline  Mirtazapine | 5-6 months  >1 month |
| 11 | Sertraline  Mirtazapine | 1 year  5 months |
| 12 | Aripiprazole  Trintellix | >1 year  4 months |
| 13 | Venlafaxine | 5 months |
| 14 | Venlafaxine | 2 years |
| 15 | Trazodone  Venlafaxine | 4 months  3 months |
| 16 | Bupropion | 8 years |
| 17 | Trintellix | 6.5 months |
| 18 | Bupropion | 3 months |
| 19 | Sertraline  Bupropion | 1.25 years  2 months |
| 20 | Trintellix | 2 months |
| 21 | Cipralex  Trazodone | >1 month  >1 month |
| ^a^Refers to the order in which participants were enrolled.  ^b^Prior to the time of the first PET scan | | |
